# Lessons learned from the use of HRP-2 based rapid diagnostic test in community-wide screening and treatment of asymptomatic carriers of *Plasmodium falciparum* in Burkina Faso

**DOI:** 10.1186/1475-2875-13-30

**Published:** 2014-01-27

**Authors:** Alfred B Tiono, Alphonse Ouédraogo, Amidou Diarra, Sam Coulibaly, Issiaka Soulama, Amadou T Konaté, Aïssata Barry, Amitava Mukhopadhyay, Sodiomon B Sirima, Kamal Hamed

**Affiliations:** 1 Centre National de Recherche et de Formation sur le Paludisme (CNRFP), 01 BP 2208, Ouagadougou 01, Burkina Faso, West Africa; 2 Novartis Healthcare Private Limited, Hyderabad 500081, India; 3 Novartis Pharmaceuticals Corporation, One Health Plaza, East Hanover, NJ 07936-1080, USA

**Keywords:** Malaria, *Plasmodium falciparum*, Asymptomatic carriers, Community screening, Rapid diagnostic tests

## Abstract

**Background:**

Rapid diagnostic tests (RDTs) are immune chromatographic tests targeting antigens of one or more *Plasmodium* species and offer the potential to extend accurate malaria diagnosis in endemic areas. In this study, the performance of *Plasmodium falciparum-*specific histidine-rich protein-2 (PfHRP-2) RDT in the detection of asymptomatic carriers from a hyperendemic region of Burkina Faso was compared with microscopy to gain further insight on its relevance in community-based interventions.

**Methods:**

The performance of HRP-2 test was evaluated in terms of sensitivity, specificity, positive and negative predictive values, discordant values, likelihood ratios, accuracy, and precision using microscopy as the 'gold standard’. This analysis was carried out in a controlled, parallel, cluster-randomized (18 clusters; 1:1) study in children and adults. The effect of systematic treatment of *P. falciparum* asymptomatic carriers during three consecutive monthly community screening campaigns on the incidence of symptomatic malaria episodes over a 12-month period was compared with no treatment of asymptomatic carriers.

**Results:**

Sensitivity of HRP-2 test in asymptomatic carriers was higher in campaign 1 (92.4%) when compared to campaign 2 (84.0%) and campaign 3 (77.8%). The sensitivity of HRP-2 test increased as parasite density increased across all the age groups. Highest sensitivity (≥97.0%) was recorded at parasite densities of 1,000-4,999/μl, except for children aged 10 to 14 years. The specificity of HRP-2 test was comparable across age groups and highest in campaign 3 (95.9%). The negative predictive values were high across the three campaigns (≥92.7%) while the positive predictive values ranged from 23.2 to 73.8%. False-positive and false-negative rates were high in campaign 1 and campaign 3, respectively.

**Conclusion:**

The performance of HRP-2 test in detecting asymptomatic carriers of *P. falciparum* varied by age and parasite density. Although the use of HRP-2 test is beneficial for the diagnosis of acute malaria, its low sensitivity in screening asymptomatic carriers may limit its utility in pre-elimination interventional settings. The use of a practical and more sensitive test such as loop-mediated isothermal amplification in combination with a cost effective HRP-2 test may be worth exploring in such settings.

## Background

Both microscopy and rapid diagnostic tests (RDTs) have been recommended by the World Health Organization (WHO) for diagnosis of malaria. The choice between the two methods varies according to available skills, patient-case load and local setting. Microscopy is the reference method for malaria diagnosis and can be used to identify species, parasite density and response to anti-malarial therapy. However, a major drawback of this tool is its requirement of well-trained, skilled staff and an energy source to power the microscope [1].

There are currently over 200 RDTs produced by 60 manufacturers, which detect *Plasmodium falciparum*, *Plasmodium vivax* or all human *Plasmodium* species [2]. Target antigens for *P. falciparum* detection are *P. falciparum-*specific histidine-rich protein-2 (PfHRP-2) and *P. falciparum-*specific parasite lactate dehydrogenase (Pf-pLDH). For *P. vivax* detection, the target antigen is *P. vivax-*specific parasite lactate dehydrogenase (Pv-pLDH). For all human *Plasmodium* species, pan-pLDH and aldolase can be detected by RDTs [3]. In field trials, malaria RDTs have demonstrated ≥90% sensitivity and specificity for *P. falciparum* infection with parasite densities of ≥200 parasites/μl [4]. HRP-2 tests are highly sensitive for *P. falciparum* infections at parasite densities above 100–200 parasites/μl, but have limited reliability at lower parasite densities [4]. Detection of *P. vivax* varies at densities of 100–200 parasites/μl and higher, depending on the RDT product and target antigen [5]. The specificity of HRP-2 tests raises concerns in areas of intense malaria transmission due to slow clearance and persistence of HRP-2 antigens in the bloodstream for several weeks due to prior infections [3,6-8]. Another recently reported concern was a high rate of false-negative results in asymptomatic carriers of *P. falciparum*. False-negative results were reported only in individuals with asymptomatic infections, suggesting that parasites that fail to produce HRP-2 can cause patent bloodstream infections [9].

Asymptomatic carriers are individuals who are infected with plasmodial asexual forms, with or without gametocytes, but do not present any clinical symptoms. These individuals are a concern because they serve as reservoirs of parasites including gametocytes that are difficult to detect, and diagnosis presents challenges due to lack of distinct clinical manifestations. There is a positive correlation between number of asymptomatic carriers and high transmission, which may be due to exposure related immunity [10].

The aim of this paper is to report HRP-2 based RDT results from a recent cluster-randomized trial in Burkina Faso, which investigated the systematic, community-wide screening and treatment of asymptomatic carriers of *P. falciparum* with artemether-lumefantrine (AL). The main outcomes of the study have already been published [11]; however, the data on the HRP-2 test performance provide an opportunity to gain further insight on its relevance in detection of *P. falciparum* asymptomatic carriers in such community-based interventions.

## Methods

### Approach for screening of asymptomatic carriers

This analysis was carried out in a controlled, parallel, cluster-randomized study to evaluate the performance of HRP-2 based RDT in comparison with microscopy in detection of *P. falciparum* asymptomatic carriers in the health district of Saponé, Burkina Faso, which is an area with marked seasonal *P. falciparum* malaria transmission from June to November. The incidence of symptomatic malaria episodes in children (<5 years) and adults over a 12-month period was compared with asymptomatic carriers who were not treated. A total of 18 clusters, each comprising one village, were selected to participate in this trial, and were randomized in a 1:1 ratio to either the intervention or control arm.

Prior to the transmission (rainy) season, screening and treatment of asymptomatic carriers took place and all inhabitants of the intervention arm and 40% of inhabitants of the control arm participated in three community screening campaigns (campaign 1 to 3), which were approximately one month apart between January and April 2011. During each screening campaign, subjects in the intervention arm were home visited and screened for *P. falciparum* asexual forms using HRP-2 test. Blood smears for delayed microscopy readings were simultaneously collected. In the control arm, only blood smears for delayed microscopy readings were obtained. RDT-positive individuals from the intervention arm received treatment with AL (20 mg artemether and 120 mg lumefantrine [Coartem®, Novartis Pharma AG, Basel, Switzerland]) or AL dispersible, twice daily for three consecutive days. As inherent to the study design, results reported here are only from the intervention arm where both HRP-2 based RDT and microscopy data were concomitantly collected.

### Rapid diagnostic test

First Response® Malaria Ag, (Premier Medical Corp Ltd., Nani-Daman, India) was used. This is a HRP-2 test recommended by WHO for its performance at low parasite densities, low false-positive rate, and high heat stability [12]. RDT stock was stored in an air-conditioned room with ambient temperature and humidity recorded twice daily at the Centre National de Recherche et de Formation sur le Paludisme (CNRFP). During the screening campaigns, in the absence of electricity, test kits were kept in cold boxes within the local health facility to which the study clusters reported. Storage temperature was recorded twice daily (morning and afternoon) by the study nurses.

### Diagnostic microscopy

Blood films were air-dried and Giemsa-stained for examination by a light microscope fitted with 100 × oil immersion lens at a single laboratory. At least 200 thick film fields were examined before a slide was declared negative. If *Plasmodium* asexual forms were found, a total of 200 thick film fields were screened for *Plasmodium* species other than *P. falciparum*. If *P. falciparum* was present, a count of asexual forms against leukocytes was made using a tally counter. Counting was conducted based on at least 200 leukocytes in consistence with WHO standards. If less than 10 parasites were identified from the 200 leukocyte screened, the count was extended to 1,000 leukocytes. On the other hand, if *P. falciparum* gametocytes were seen, a gametocyte count was performed against 1,000 leukocytes. All slides were read by two independent microscopists. If the ratio of densities from the first two readings was >1.5 or < 0.67, or if less than 30 parasites were counted with an absolute difference of more than 10 in the number of parasites, the slide was evaluated by a third microscopist. The definitive result was the mean of the parasite density of the two most concordant readings. Microscopist competency was evaluated through two external quality control (EQC) programmes. The first EQC was carried out by the College of American Pathology proficiency testing and included a set of five slides provided to each microscopist for reading; three rounds of proficiency testing were conducted per year. The second EQC was performed by WHO (National Institute for Communicable Diseases) and involved the reading of a set of 20 slides every quarter by each microscopist. Only those with a score of at least 80%, graded as ‘competent’, were involved in the reading of trial participants’ slides.

### Statistical methods

Data were entered into an electronic web-based case report form and were analysed using SAS® version 9.2 on UNIX according to a pre-defined analytical plan. The study sample was categorized into four age groups: zero to four years, five to nine years, 10 to 14 years, and ≥15 years. Six categories of parasite densities were considered for subjects with parasitaemia: 0–99 parasites/μl, 100–249 parasites/μl, 250–499 parasites/μl, 500–999 parasites/μl, 1,000-4,999 parasites/μl, and ≥5,000 parasites/μl.

Each HRP-2 result was categorized as true positive (TP), true negative (TN), false positive (FP), or false negative (FN). Sensitivity was calculated as TP/(TP + FN) and specificity as TN/(TN + FP). Positive predictive value (PPV) was defined as TP/(TP + FP) and negative predictive value (NPV) as TN/(TN + FN). False-positive rate was calculated as FP/(FP + TN) and false-negative rate was calculated as 1-(TN/[TN + FP]). Positive likelihood ratio was defined as the probability of a positive test result in patients with the disease divided by the probability of a positive test result in patients without the disease (i.e., sensitivity/[1 - specificity]), and negative likelihood ratio as the probability of a negative test result in patients with the disease divided by the probability of a negative test result in patients without the disease (i.e., [1 - sensitivity]/specificity). Accuracy, the proportion of all tests that yielded a correct result, was defined as (TP + TN)/(TP + TN + FP + FN). Cohen’s Kappa value, the quantitative measure of the magnitude of agreement between two tests was calculated. The test performance was evaluated across the three screening campaigns and for categories of age and parasite density. Geometric mean parasite density by age group was also reported.

### Ethical aspects

The clinical trial protocol and informed consent forms were approved by the CNRFP Institutional Review Board and by the National Ethical Committee for Health Research of Burkina Faso. Prior to study initiation, a community meeting was held in each of the selected clusters to discuss the study objectives with the communities. The freedom of each individual household and household member to decide on study participation was discussed to minimize the potential influence of key opinion leaders in each cluster. Individual informed consent was obtained from each participant during a visit to the household prior to any study procedure.

## Results

**Figure 1:**
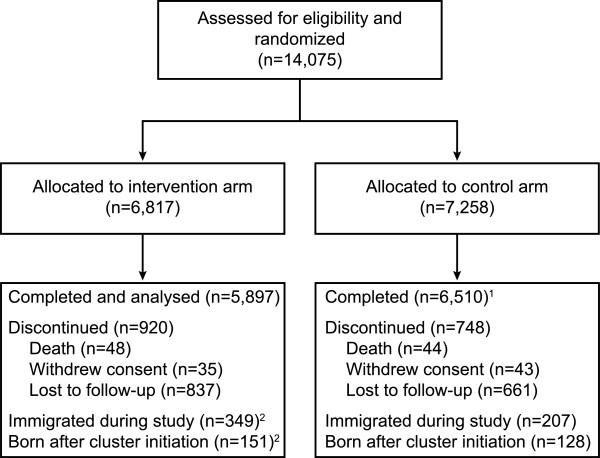
**Consort chart. **^1^RDT was not done for subjects in the control arm and participants were excluded from the analysis. ^2^Not included in analysis as assessment was done after third campaign.

Of 14,075 subjects assessed for eligibility, a total of 6,817 subjects in the intervention arm and 7,258 subjects in the control arm were recruited and enrolled in the study (Figure 1). The intervention and control arms were similar in terms of demographic characteristics with the exception of ethnicity; the intervention arm had a higher proportion of Fulani. The primary outcomes of this study were reported by Tiono *et al.*[11].

A total of 17,505 samples from 6,817 subjects in the intervention arm were examined by microscopy and HRP-2 test across the three campaigns (Table 1). About 47% of the participants were women and their mean age at baseline was 24.4 years.

**Table 1:** Characteristics of study participants across three screening campaigns

	**Campaign 1**	**Campaign 2**	**Campaign 3**
**Number of participants**	5,633	5,718	6,154
**Mean age (years)**	24.4	23.9	23.0
**Number (%) of females**	2,617 (46.5)	2,664 (46.6)	2,863 (46.5)
**Prevalence of *P. falciparum *** **infection (by microscopy), n (%)**			
0- <5	689 (12.2)	205 (3.6)	193 (3.1)
5-9	971 (17.2)	318 (5.6)	271 (4.4)
10-14	841 (14.9)	253 (4.4)	218 (3.5)
≥15	1,080 (19.1)	294 (5.1)	230 (3.7)
Overall	3,581 (63.6)	1,070 (18.7)	912 (14.8)
**Prevalence of *P. falciparum *** **infection (by HRP-2 test), n (%)**			
0- <5	606 (10.8)	238 (4.1)	86 (1.4)
5-9	734 (13.0)	175 (3.0)	52 (0.8)
10-14	674 (12.0)	114 (2.0)	49 (0.8)
≥15	1,060 (18.8)	336 (5.9)	190 (3.1)
Overall	3,074 (54.6)	863 (15.1)	377 (6.1)
**Frequency of *P. falciparum *** **infection by density cut-offs (parasites/μl), n (%)**			
1-99	798 (22.3)	272 (25.4)	258 (28.3)
100-499	1,013 (28.3)	370 (34.6)	301 (33.0)
500-999	599 (16.7)	182 (17.0)	148 (16.2)
1,000-4,999	877 (24.5)	199 (18.6)	165 (18.1)
≥5,000	294 (8.2)	47 (4.4)	40 (4.4)
Overall	3,581 (100.0)	1,070 (100.0)	912 (100.0)
**Geometric mean *P. falciparum *** **density in positives by age (parasites/μl)**			
0- <5	1,348	702	756
5-9	795	482	387
10-14	406	273	241
≥15	168	127	111
Overall	470	315	291

Age was considered at cluster initiation. Percentages in prevalence subgroups are based on the total number of subjects in the respective subgroup.

Overall, 5,563 (31.8%) samples were detected to be positive using microscopy (Table 1). The prevalence of *P. falciparum* infection as detected by microscopy was highest among subjects aged ≥15 years, followed by five to nine year olds and 10 to 14 year olds. Using HRP-2 test, 4,314 (24.6%) samples were detected to be positive for *P. falciparum* (Table 1)*.* The prevalence of *P. falciparum* infection by age as detected by HRP-2 test followed the same trend as microscopy in campaign 1, but in campaigns 2 and 3, prevalence was highest among subjects aged ≥15 years, followed by <5 year olds and 10 to 14 year olds. The prevalence of *P. falciparum* infection by density ranged from 8.2 to 28.3% in campaign 1, 4.4 to 34.6% in campaign 2, and 4.4 to 33.0% in campaign 3 (Table 1). Across the three campaigns, the geometric mean *P. falciparum* density decreased with increasing age; it was highest in children <5 years of age (Table 1).

**Figure 2:**
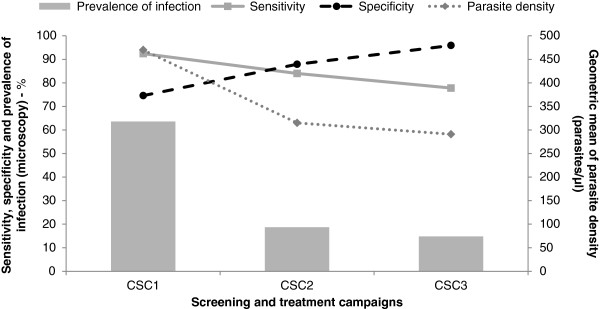
**Prevalence of *Plasmodium falciparum *** **infection, parasite count and rapid diagnostic test accuracy (sensitivity and specificity) according to screening campaigns.**

Sensitivity of HRP-2 test in asymptomatic carriers was higher in campaign 1 when compared to campaign 2 and campaign 3 (Table 2 and Figure 2). Specificity of HRP-2 test was highest in campaign 3 (Table 2 and Figure 2). Negative predictive values were high across the three campaigns, while positive predictive values ranged from 23.2 to 73.8%. An overview of the discordant results (i.e., either a false-positive or false-negative HRP-2 compared with microscopy) is given in Table 2. False-positive and false-negative rates were high in campaign 1 and campaign 3, respectively. Positive likelihood ratio was highest in campaign 3 followed by campaign 2 and campaign 1. Negative likelihood ratio was lowest in campaign 1, followed by campaign 2 and campaign 3. The proportion of all true HRP-2 test results yielded an accuracy of 0.82, 0.88, and 0.95 in campaigns 1, 2 and 3, respectively. The agreement between the two techniques, HRP-2 test and microscopy, was highest in campaign 1.

**Table 2:** Overall performance of HRP-2 test in asymptomatic carriers across three screening campaigns

	**Campaign 1**	**Campaign 2**	**Campaign 3**
**Sensitivity, % (95% CI)**	92.4 (91.4, 93.5)	84.0 (79.3, 88.6)	77.8 (71.5, 84.0)
**Specificity, % (95% CI)**	74.6 (73.1, 76.1)	87.9 (87.0, 88.7)	95.9 (95.4, 96.4)
**Positive predictive value, % (95% CI)**	73.8 (72.2, 75.3)	23.2 (20.3, 26.0)	35.4 (30.5, 40.2)
**Negative predictive value, % (95% CI)**	92.7 (91.7, 93.7)	99.2 (99.0, 99.5)	99.3 (99.1, 99.5)
**False-positive rate**	0.25	0.12	0.04
**False-negative rate**	0.08	0.16	0.22
**Positive likelihood ratio**	3.64	6.92	19.01
**Negative likelihood ratio**	0.10	0.18	0.23
**Accuracy**	0.82	0.88	0.95
**Kappa, % (95% CI)**	65.2 (63.2, 67.1)	31.9 (28.3, 35.4)	46.6 (41.4, 51.8)

Generally, the sensitivity of HRP-2 test increased as parasite density increased across all the age groups (Table 3 and Figure 2). At parasite densities of 1,000-4,999/μl, HRP-2 test recorded the highest sensitivity, except for children aged 10 to 14 years.

**Table 3:** Performance of HRP-2 test in asymptomatic carriers by age and parasite density

**Age group (years)**	**Parasite density (/μl)**	**N**	**Positive**	**Negative**	**Sensitivity % (95% CI)**	**Specificity % (95% CI)**
**0- <5**	0-99	2,663	433	2,230	74.6 (64.5, 84.8)	
	100-499	90	83	7	92.2 (86.7, 97.8)	
	500-999	86	85	1	98.8 (96.6, 100)	
	1,000-4,999	211	209	2	99.1 (97.7, 100)	
	≥5,000	117	114	3	97.4 (94.6, 100)	
	Overall	3,167	924	2,243	94.6 (92.8, 96.5)	85.3 (84.0, 86.7)
**5-9**	0-99	2,147	327	1,820	84.0 (75.7, 92.3)	
	100-499	184	171	13	92.9 (89.2, 96.6)	
	500-999	145	143	2	98.6 (96.7, 100)	
	1,000-4,999	246	245	1	99.6 (98.8, 100)	
	≥5,000	69	68	1	98.6 (95.7, 100)	
	Overall	2,791	954	1,837	96.0 (94.5, 97.4)	87.3 (85.8, 88.7)
**10-14**	0-99	2,124	346	1,778	85.5 (79.8, 91.2)	
	100-499	215	206	9	95.8 (93.1, 98.5)	
	500-999	104	103	1	99.0 (97.2, 100)	
	1,000-4,999	145	144	1	99.3 (98.0, 100)	
	≥5,000	31	31		100 (100, 100)	
	Overall	2,619	830	1,789	95.0 (93.3, 96.7)	88.8 (87.4, 90.2)
**≥15**	0-99	8,287	1,129	7,158	73.1 (68.8, 77.5)	
	100-499	310	265	45	85.5 (81.6, 89.4)	
	500-999	107	101	6	94.4 (90.0, 98.8)	
	1,000-4,999	66	64	2	97.0 (92.8, 100)	
	≥5,000	17	10	7	58.8 (35.4, 82.2)	
	Overall	8,787	1,569	7,218	81.4 (78.8, 83.9)	89.4 (88.7, 90.1)

N = number of subjects for whom RDT and microscopy results were available.

## Discussion

This paper describes the performance of HRP-2 test in comparison with microscopy in the detection of asymptomatic carriers in community-wide screening and treatment campaigns from a hyperendemic region of Burkina Faso. The findings provide evidence that while HRP-2 test might be a convenient diagnostic tool in a similar community based intervention with highly demanding logistics, it has obvious limitations that could jeopardize the success of the intervention.

### What lessons were learned from this analysis?

Two main lessons could be drawn from the results of this trial. The first lesson is that HRP-2 test might not be suitable to use across all age groups in screening campaigns for asymptomatic carriers. Indeed, the test sensitivity decreased with age. This finding is consistent with previous reports showing that the proportion of infected individuals with low parasite densities increased with age, most likely due to age acquired immunity [13-15]. Thus, the age-dependent immune status might have lowered the HRP-2 test sensitivity [16]. This reduced sensitivity might have a serious impact on the effectiveness of mass screening and treatment (MSAT) strategies aiming to interrupt transmission. Indeed, as shown in a previous study, although gametocytes are most commonly detected in children, the proportion of asexual parasites that will develop into gametocytes may increase with age indicating the potential enormous contribution of adults for human infectious reservoir of malaria [17]. Another study that evaluated the contribution of different age groups to the human infectious reservoir found that adults may be responsible for 28 to 38% of mosquito infections [18]. This proportion could be even larger if differences in body size and exposure index to mosquitoes between adults and children were considered [19]. In total, failing to detect asexual parasites in adults in MSAT studies would thus highlight the missed opportunity in clearing the probably most important reservoir of parasites.

Overall, the HRP-2 test specificity was low and increased with age. Difference was only statistically significant when comparison was done between zero to <5 years and both 10 to 14 and ≥15 years age groups. Previous studies showed that recently treated infections may lead to false-positive results [3,6-8]. The delayed clearance and persistence of HRP-2 antigen in the blood, even after complete eradication of parasites, can limit the utility of the test [6,14,20]. The difference between age groups could be explained by the difference in incidence of clinical malaria episodes. Younger children are more prone to developing clinical symptoms and getting treated. Hence, the likelihood of detecting HRP-2 antigenaemia post treatment is high in this subpopulation. In theory, there is no potential impact of this low specificity on the effectiveness of the MSAT intervention. However, frequent false-positive findings can lead to unnecessary treatment [20].

The second important lesson from this trial is that HRP-2 might not be sufficient as a single diagnostic method in MSAT involving multiple screening campaigns. While the sensitivity and positive predictive values were high at the first campaign, a significant decrease below recommended standard by WHO [21] was observed during the next two campaigns. This trend was similar to the one observed for the prevalence of infection, and the geometric mean parasite density (Figure 2). A possible explanation is that several subjects may have harboured parasites at low densities that were below detection limit of the HRP-2 test. The proportion of subjects with parasite count <500 parasites/μl rose from 50.6% at first campaign to ~60% at second and third campaigns. This is supported by similar findings in Solomon Islands (low transmission setting) where a study conducted showed that a large proportion of asymptomatic *P. falciparum* and *P. vivax* infections had low and submicroscopic parasite densities that were not detected by RDT. The results suggested that the detection limit of the ICT combo kit was ~100 parasites/μl for *P. falciparum* and >300 parasites/μl for *P. vivax*[13]. Another study evaluated the performance of Optimal® rapid malaria test compared to expert microscopy as a tool for detecting asymptomatic malaria in an area of Thailand that is endemic for both *P. falciparum* and *P. vivax*. The low sensitivity of Optimal® rapid malaria test along with poor assay specificity and high number of false-positive cases in individuals with parasite densities <500/μl, suggested that the majority of malaria cases may not have been accurately detected during a similar surveillance programme [22].

Contrary to the sensitivity trend, the specificity and negative predictive values increased as the screening campaigns progressed. This provides evidence that the use of HRP-2 test has made it possible to comply with the overall goal of the MSAT intervention, which was to treat only those carrying malaria parasites, therefore minimizing overuse of drugs in non-parasitaemic subjects, and in turn reducing drug pressure.

### Are there alternatives to RDTs in such trials?

Microscopy is widely accepted as a gold standard laboratory method. However, examining blood smears with high quality and accuracy requires extensive experience and training [23]. Furthermore, the use of microscopy may not be feasible in remote areas where malaria is prevalent [24] and results may not be readily available for treatment decision making. The experience in this trial suggests that a point-of-care malaria diagnostic tool is the best option to minimize logistical hurdles and ensure good coverage of the population. To overcome the limitations of detecting infections of lower density by microscopy and RDT, molecular diagnostic methods, such as polymerase chain reaction (PCR) have been developed. Studies using PCR in low transmission settings have revealed a high proportion of low-density parasitaemias and asymptomatic carriers that were not detected by RDT or microscopy [5,13,25,26].

Loop mediated isothermal amplification (LAMP) is an innovative gene amplification technique emerging as a simple rapid diagnostic tool for early detection and identification of microbial diseases. There is evidence that LAMP may be a fast, simple, and cost-effective diagnostic approach in detecting malaria parasites. LAMP uses DNA polymerase and amplifies a target DNA sequence with high sensitivity and specificity under isothermal conditions. Several studies have evaluated the sensitivity and specificity of LAMP for the detection and differentiation of *Plasmodium* species for human malaria. An advantage of LAMP is that results can be analysed with the naked eye based on turbidity or the use of fluorescent dyes [27]. Studies have also shown that LAMP is superior to expert microscopy, has diagnostic accuracy similar to PCR, and can produce results in a shorter time [28-30]. Current results and further development of LAMP may provide the possibility of its future use in the diagnosis of malaria.

### What are possible scenarios for similar future interventions?

RDTs are still appealing since they are easy to use and can help overcome the challenges of microscopy in field-based trials. However, low sensitivity of RDTs in screening asymptomatic carriers limits their utility as single diagnostic tool in interventions targeting the parasite reservoirs (i.e., MSAT). While LAMP may be an option, given its ease of use in the field and diagnostic accuracy, its role in MSAT trials needs to be explored especially in settings where prevalence of malaria is high. It might not be cost effective to use this tool as stand-alone in such settings. Rational combinations with RDTs need to be explored. One possible scenario would be to use both RDT and LAMP in the first screening and treatment campaign, where RDT is used in children and LAMP in individuals ≥15 years. This approach adds cost and complexity to the trial implementation, but could help to prevent spread of the infection (by those who were not detected by RDT) in the interval before the next campaign. In subsequent campaigns, using LAMP in newcomers to the area or in the whole population, based on malaria transmission dynamics, may be targeted.

In areas with reports of high RDT sensitivity that is consistent across ages, exclusive use of RDT at the first campaign could be a better option to minimize cost. LAMP should be preferred for subsequent campaigns. Computer simulation analyses might be necessary to explore these scenarios and other possible ones to identify cost-effective solutions for better decision making.

## Conclusion

The performance of HRP-2 test in *P. falciparum* asymptomatic carriers varied by age and parasite density. This low sensitivity in screening asymptomatic carriers may limit its utility in pre-elimination interventional settings. LAMP is a practical and more sensitive test; but cost may be a barrier to its use as single diagnostic tool in large-scale MSAT trials. Cost effective combination of HRP-2 tests and LAMP may be worth exploring.

## Competing interests

ABT and AO have received honoraria from Novartis Pharma AG, Basel, Switzerland to attend Advisory Board meetings to discuss this study and manuscript. AM is an employee of Novartis Healthcare Private Limited. KH is an employee of Novartis Pharmaceuticals Corporation. AD, SC, IS, ATK, AB, and SBS declared no competing interests.

## Authors’ contributions

ABT, AO, AD, SC, AM, SBS, and KH were involved in the design of the study, data interpretation, and defining the content for the manuscript. ABT, AO, AD, SC, IS, ATK, AB, and SBS were involved in data collection, while ABT, AO, AM, and KH conducted the data analysis. ABT, AM and KH were involved in writing the manuscript. All of the authors had full access to data in the study, discussed the results, critically reviewed the draft manuscript, and agreed on the final version. ABT, the corresponding author, had final responsibility for the decision to submit the manuscript for publication. Editorial assistance was provided by Ubhayabharathi Gurunath and Krishna Swetha Gummuluri, professional medical writers (Novartis Healthcare Private Limited).
